# Performance changes and relationship between vertical jump measures and actual sprint performance in elite sprinters with visual impairment throughout a Parapan American games training season

**DOI:** 10.3389/fphys.2015.00323

**Published:** 2015-11-06

**Authors:** Irineu Loturco, Ciro Winckler, Ronaldo Kobal, Cesar C. Cal Abad, Katia Kitamura, Amaury W. Veríssimo, Lucas A. Pereira, Fábio Y. Nakamura

**Affiliations:** ^1^Nucleus of High Performance in SportSão Paulo, Brazil; ^2^Brazilian Paralympic CommitteeBrasília, Brazil; ^3^Department of Physical Education, State University of LondrinaLondrina, Brazil

**Keywords:** Paralympics, track and field, muscle power, physical disability, blind athletes

## Abstract

The aims of this study were to estimate the magnitude of variability and progression in actual competitive and field vertical jump test performances in elite Paralympic sprinters with visual impairment in the year leading up to the 2015 Parapan American Games, and to investigate the relationships between loaded and unloaded vertical jumping test results and actual competitive sprinting performance. Fifteen Brazilian Paralympic sprinters with visual impairment attended seven official competitions (four national, two international and the Parapan American Games 2015) between April 2014 and August 2015, in the 100- and 200-m dash. In addition, they were tested in five different periods using loaded (mean propulsive power [MPP] in jump squat [JS] exercise) and unloaded (squat jump [SJ] height) vertical jumps within the 3 weeks immediately prior to the main competitions. The smallest important effect on performances was calculated as half of the within-athlete race-to-race (or test-to-test) variability and a multiple regression analysis was performed to predict the 100- and 200-m dash performances using the vertical jump test results. Competitive performance was enhanced during the Parapan American Games in comparison to the previous competition averages, overcoming the smallest worthwhile enhancement in both the 100- (0.9%) and 200-m dash (1.43%). In addition, The SJ and JS explained 66% of the performance variance in the competitive results. This study showed that vertical jump tests, in loaded and unloaded conditions, could be good predictors of the athletes' sprinting performance, and that during the Parapan American Games the Brazilian team reached its peak competitive performance.

## Introduction

In general, the public is astonished by the performance of Paralympic athletes, given their extreme physical and technical capacities in spite of the presence of mild to severe physical disabilities. Although it is known that sprinting performance in Paralympic Track and Field has improved at a higher rate than the Olympic results (Grobler et al., 2015), there has been no systematic analysis of the variability and progression in competitive performance of successful teams in the months of preparation leading up to a main competition (e.g., Parapan American Games).

It has been suggested that the smallest important effect in performance at a target international event is one-half of the typical within-athlete random variability between events (Hopkins et al., 1999). An important performance progression to enhance the chances of a medal for Olympic and Paralympic swimmers in the year leading up to the main competition has been estimated to be ≈1–2% (Pyne et al., 2004; Fulton et al., 2009). In addition, in elite Olympic track athletes (including sprinters), an improvement of as little as 0.3–0.5% is considered meaningful (Hopkins, 2005). Calculating the smallest important change in Paralympic sprinters may help coaches to define targets for performance improvements in the year of preparation for the upcoming Paralympic Games.

Importantly, although the actual performance is considered the “gold standard” to assess elite athletes, simple and field based tests assessing key components of competitive outcomes can be considered as important evaluation and monitoring tools. For instance, it has been shown that loaded and unloaded vertical jumping performances are largely correlated with sprinting speed in elite sprinters (Loturco et al., 2015a,b). Confirmation of these associations between loaded/unloaded jumps (or even in combination) and sprinting performance in Paralympic athletes may help coaches to choose appropriate tests in order to evaluate and monitor Paralympic sprinters. This is particularly relevant to athletes with visual impairments as vertical jumping tests may be executed without the need for much assistance, enabling easy implementation in the training routines.

In this regard, it is important to know the within-athlete variability and progression in vertical jumping performance, to allow coaches to effectively decide whether a given change in testing performance might be considered meaningful or within the trivial variation caused by biological and/or technical factors. Based on this information, coaches can better select training strategies to optimize athletes' sports form, without necessarily assessing the actual competitive performance. Furthermore, simple tests can be used on a daily basis, thus providing fine feedback for training adjustments.

Therefore, the aim of this study was to estimate the magnitude of variability and progression in actual competitive and field vertical jump test performances in elite Paralympic sprinters with visual impairment in the year leading up to the 2015 Parapan American Games. Furthermore, we aimed to investigate the relationships between loaded and unloaded vertical jumping test results and the actual competitive sprinting performance, through the use of simple and multiple linear regression analyses.

## Materials and methods

Seven official competitions were analyzed (four national, two international and the Parapan American Games 2015) between April 2014 and August 2015. The competitions were sanctioned by the Brazilian Paralympic Committee (CPB), International Paralympic Committee (IPC), and Americas Paralympic Committee (APC), respectively. The physical assessments were performed close to the competitions (up to 3 weeks prior to the main competitions) in five different periods (between April 2014 and July 2015) scheduled by the High Performance Programs of CPB, as part of the athletes' monitoring during the competitive season. Fifteen Brazilian Paralympic sprinters with visual impairment, from 18 to 36 years old, took part of the study (seven men and eight women, classes: T11 [*n* = 9]; T12 [*n* = 3]; and T13 [*n* = 3]). All athletes were part of the permanent Brazilian team, frequently involved in national and international competitions. This elite sample comprised four world champions, two Paralympic champions, four world record holders, two Paralympic record holders, eight world medalists, 11 Paralympic medalists, 12 top-five athletes and two top-ten athletes in the 2015 world ranking, thus attesting their high level of competitiveness.

For analysis purposes, only the times attained in the finals were retained. Across the seven competitions, a total of 120 official times (68 from the 100-m and 52 from the 200-m dash) were included in the analyses. Additionally, 192 test results in the five different periods using loaded (mean propulsive power [MPP] in jump squat [JS] exercise) and unloaded (squat jump [SJ] height) vertical jumps were analyzed. All the athletes had been previously familiarized with the testing procedures. This study was approved by the Ethics Committee of the Bandeirantes Anhanguera University. Prior to study participation, all the athletes signed an informed consent form.

### Vertical jumping ability

Vertical jumping ability was assessed using SJ. To perform the SJ, a static position with a 90° knee flexion angle was maintained for 2-s before every jump attempt. No preparatory movement was allowed and an experienced researcher visually inspected for proper technique. Five attempts were performed interspersed by 15-s intervals. All attempts were executed with the hands on the hips. The jumps were performed on a contact platform (Smart Jump; Fusion Sport, Coopers Plains, Australia) with the obtained flight time (t) being used to estimate the height of the rise of the body's center of gravity (h) during the vertical jump (i.e., h = gt^2^/8, where *g* = 9.81 m·s^−2^. The best attempt was used for data analysis purposes. The athletes executed the attempts without assistance.

### Bar mean propulsive power in jump squat

Bar MPP in the JS exercise was assessed on a customized Smith machine (adapted by Hammer Strength, Rosemont, IL, USA). The athletes were instructed to execute three repetitions at maximal velocity for each load, starting at 40% of their body mass (BM). The athletes with visual impairment executed a knee flexion until the thigh was parallel to the ground (≈100° knee angle for 2-s) and, after a command, jumped as fast as possible without losing contact between their shoulder and the bar. A load of 10% BM was gradually added in each set until a decrease in MPP was observed. A 5-min interval was provided between sets. All athletes attained their maximum values of MPP during the execution of the tests, within 4–5 attempts. Of note, the athletes achieved their highest MPP outputs at a load corresponding to ≈100% BM. To determine MPP, a linear transducer (T-Force, Dynamic Measurement System; Ergotech Consulting S.L., Murcia, Spain) was attached to the Smith machine bar. The finite differentiation technique was used to calculate bar velocity (Sanchez-Medina et al., 2010). As Sanchez-Medina et al. (2010) demonstrated that mean mechanical values during the propulsive phase better reflect the differences in the neuromuscular potential between two given individuals, MPP rather than peak power was used in the JS. The bar maximum MPP value obtained was considered for data analysis purposes. In order to consider the differences in BM between the athletes and avoid misinterpretation of the power outputs, these values were normalized by dividing the absolute power value by the BM (i.e., relative power = W.kg^−1^) (MPP REL). All the tests were performed by the athletes with no assistance.

### Statistical analysis

The normality of data was confirmed using the Shapiro-Wilk test. Data are presented as means ± standard deviations (SD). The smallest important effect on performances was calculated as half of the within-athlete race-to-race (or test-to-test) variability (Hopkins et al., 1999). Additionally, the within-subject coefficient of variation (CV), with 90% confidence intervals (CI), was calculated as a measure of competitive and testing performance variability. A Pearson product-moment coefficient of correlation was used to analyze the relationships between SJ and MPP REL in the JS and sprinting time in the 100- and 200-m. The vertical jumping tests took place in close proximity to five of the seven competitions. A multiple regression analysis was performed using the vertical jump test results as predictors of 100- and 200-m dash performances. The possibility of collinearity between the predictive variables in multiple regression models was examined using variance inflation factor (VIF) and tolerance (i.e., VIF < 10 and tolerance > 0.2; Kennedy, 1992; Hair et al., 1995; Tabachnick and Fidell, 2001). Intraclass correlation coefficients (ICCs) were used to indicate the relationship within SJ and JS for, respectively, jumping height and MPP. The significance level was set as *P* < 0.05.

## Results

**Figure 1:**
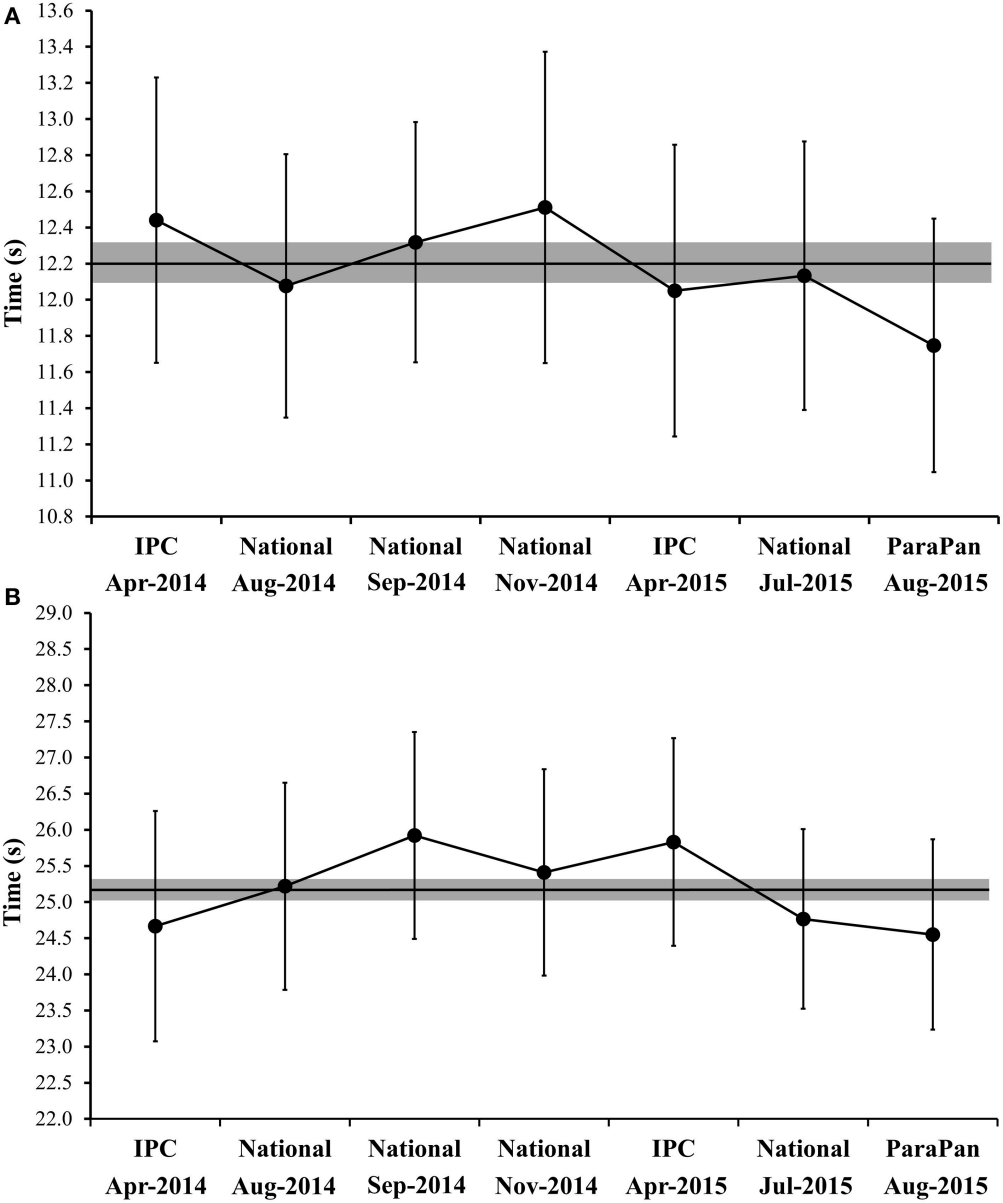
**Variation in 100- (A) and 200-m (B) dash performances across seven official competitions**. The black line represent the mean individual performances, and the gray area represent the smallest important effect on performances (i.e., calculated as half of the within-athlete race-to-race variability). *National* corresponds to competitions organized by the local Paralympic Committee; *IPC* corresponds to international competitions organized by the International Paralympic Committee; *ParaPan* corresponds to the ParaPan American Games.

**Figure 2:**
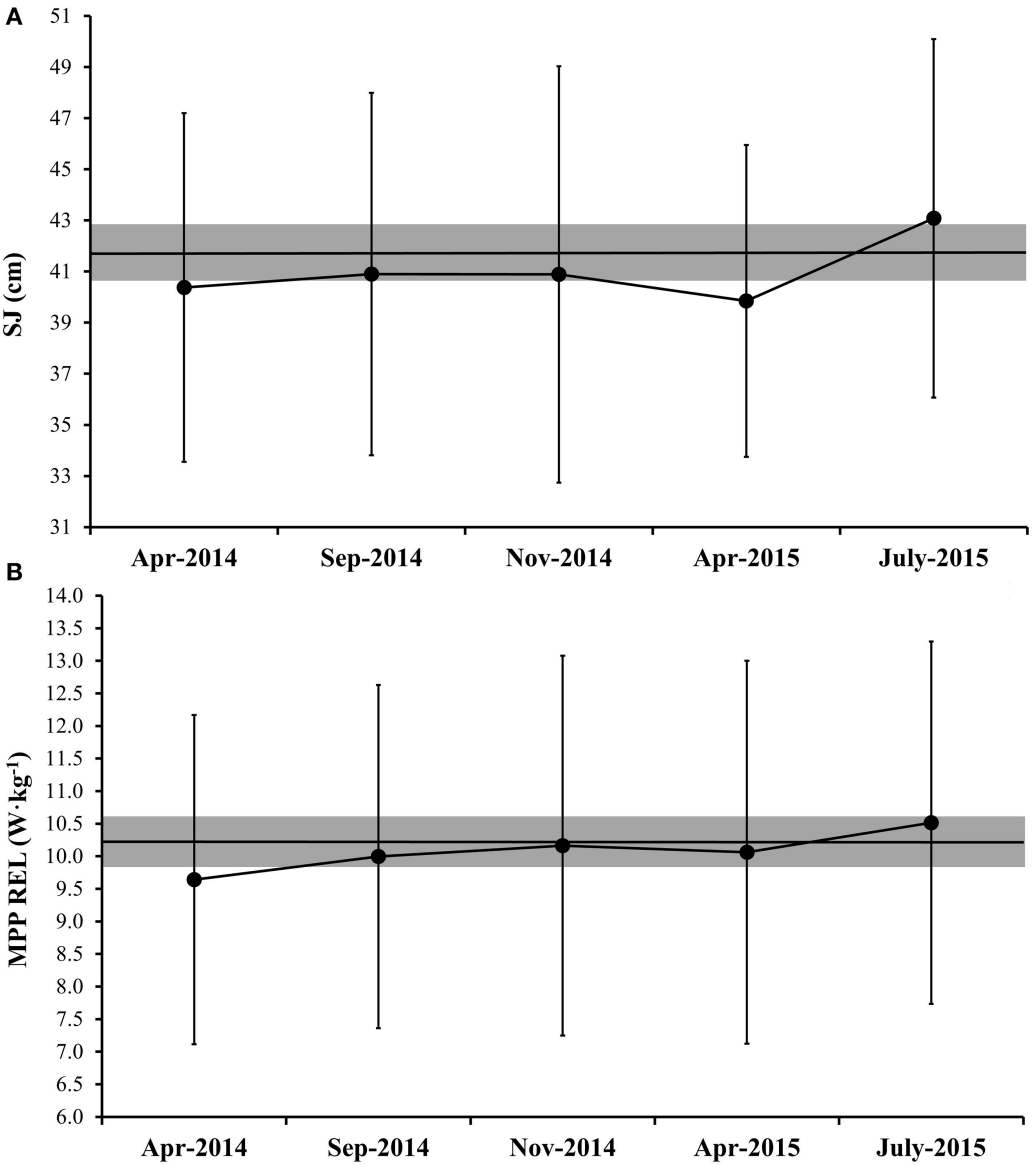
**Variation in squat jump (SJ) (A) and relative mean propulsive power in jump squat exercise (MPP REL JS) (B) test results across the five periods**. The black line represents the mean individual performances, and the gray area represents the smallest important effect on performances (i.e., calculated as half of the within-athlete test-to-test variability).

All data presented herein showed normal distribution. The VIF and tolerance values were 2.43 and 0.42, respectively, attesting that the independent variables (i.e., SJ and JS) were not collinear. The characteristics of the subjects are presented in the Table 1. The BM of the athletes did not vary substantially across the five evaluation moments (varying from 61.41 ± 9.76 to 63.84 ± 10.66). Figure 1A displays the means of the 100-m dash performances in the seven competitions. The mean CV (90% CI) over the competitions was 1.79% (1.47; 2.24). Meanwhile, Figure 1B depicts the mean performance in the 200-m events. The mean CV (90% CI) across the seven competitions was 1.35% (0.83; 1.88). The performance variation in the SJ and JS over the five moments is displayed in Figures 2A,B, respectively. Importantly, the ICC was 0.94 for the SJ and 0.92 for the loaded JS. The mean CVs (90% CI) for SJ and JS were 5.58% (4.18; 6.99) and 7.97% (5.91; 10.02), respectively.

**Table 1:** **Characteristics of the subjects (mean ± SD)**.

**Age (years)**	**Weight (kg)**	**Height (cm)**
26.5 ± 6.2	63.3 ± 10.6	169 ± 0.9

**Figure 3:**
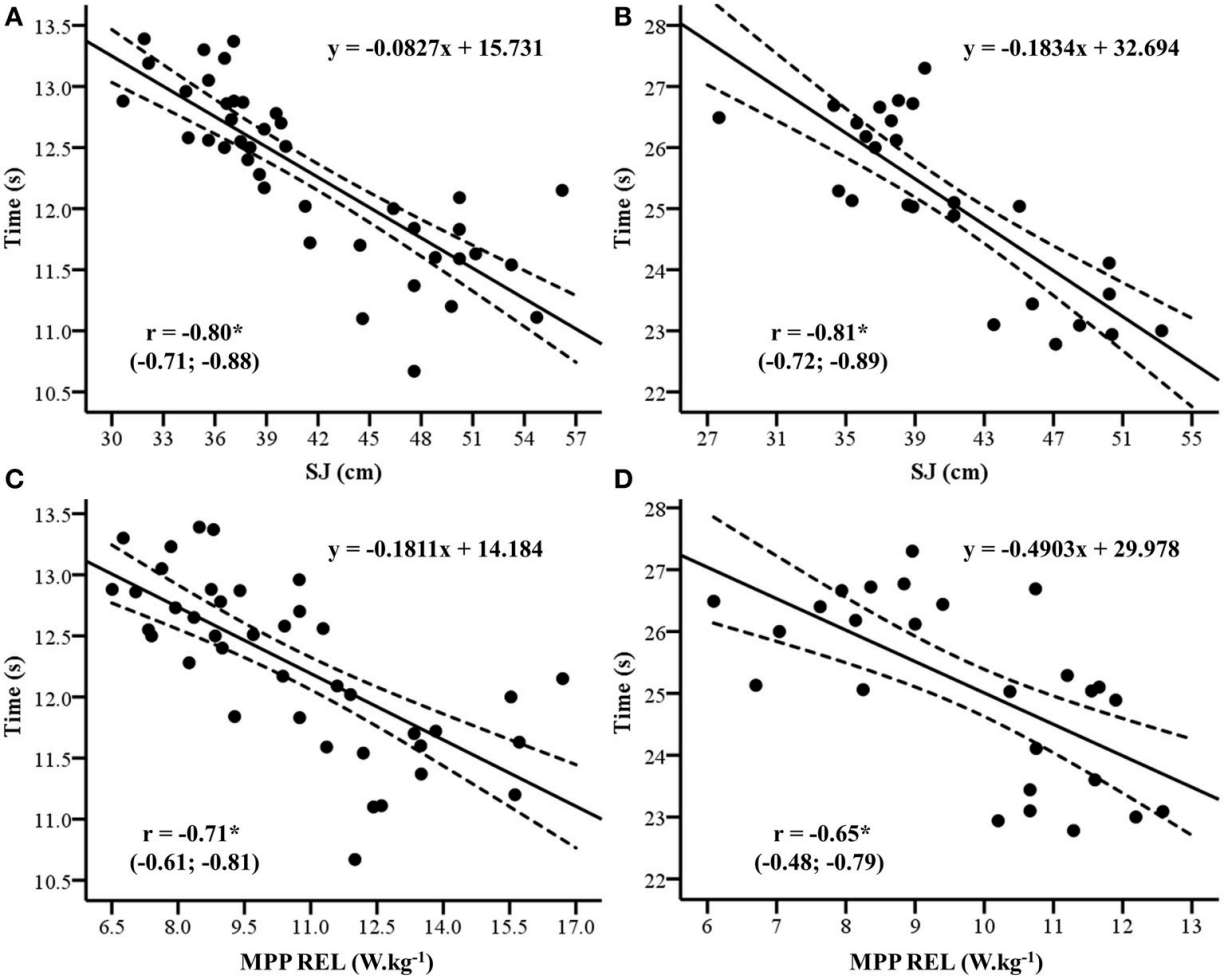
Linear regression between 100 (A,C) and 200-m (B,D) dash performances and the squat jump (SJ) height and relative mean propulsive power (MPP REL) in the jump squat (JS) exercise; ^*^P < 0.01.

The correlations (90% CI) between SJ and JS test results and 100- and 200-m dash performances were −0.80 (−0.71; −0.88) and −0.71 (−0.61; −0.81) for SJ and JS with 100-m, respectively, and; −0.81 (−0.72; −0.89) and −0.65 (−0.48; −0.79) for SJ and JS with 200-m, respectively (*P* < 0.01 for all correlations; Figure 3). The multiple regression analysis using SJ and JS test results as predictors of 100- and 200-m dash performances is presented in Table 2. The SJ and JS explained 66% of the performance variance in both 100- and 200-m dash performances.

**Table 2:** **Predictions of 100- and 200-m dash performances using multiple regression analysis**.

	***R*^2^**	**Equation**
100-m	0.66^*^	*y* = 15.558 – (0.063 × SJ) – (0.061 × JS)
200-m	0.66^*^	*y* = 32.918 – (0.167 × SJ) – (0.098 × JS)

SJ, squat jump; JS, jump squat;

* *P < 0.01*.

## Discussion

This is the first study to investigate the relationships between loaded/unloaded vertical jump tests and the actual performance obtained by top-level Paralympic sprinters in 100 and 200 m dash events. The main finding reported herein is that, providing they are executed only a few weeks before the official tournaments (i.e., from 1 to 3 weeks), SJs and loaded JSs—when combined in a multiple linear regression model—can be good predictors of the actual competitive performance of top-level athletes with visual impairment. In addition, during a given training period, the dynamics of the performance in these specific jump tests seem to be analogous to the dynamics of the actual results obtained in 100 and 200 m dash events.

Another study has already reported strong correlations between sprinting performance (i.e., measured in time) and SJ height (*r* = −0.82) in elite sprinters who compete in 100 m dash events (Loturco et al., 2015b). This “close relationship” can be explained when analyzing the mechanical aspects of unloaded SJs. In order to jump higher, an athlete has to apply a substantial amount of force against the ground—and against his/her own BM (Loturco et al., 2015a,b). Thus, this measurement is already normalized by the subject's weight, being able to express his/her relative neuromuscular potential (i.e., relative values of muscle force and muscle power; Bosco et al., 1983; Copi et al., 2014). Furthermore, from a mechanical perspective, athletes with better performances in vertical jumps are possibly more efficient at overcoming the inertia and accelerating their bodies vertically (Bunton et al., 1993; Loturco et al., 2015b). Importantly, as the ground reaction forces increase, the vertical jump height increases (Loturco et al., 2015b). The same occurs in sprinting, during the transition from lower to higher velocities (i.e., top-speed sprinting), which results in shorter support phases with simultaneous increases in vertical peak force (Nilsson and Thorstensson, 1989). Curiously, even in sprinters with visual impairment, this mechanical similarity seems to be capable of influencing the specific performance in 100 and 200 m dash events. Certainly, the user-friendly characteristic and practicality of SJ facilitates its execution by athletes with visual impairment, thus reinforcing its use as a tool to control/monitor the variation in sprinting performance presented by this specific group of Paralympics.

Loaded JSs are usually performed with moderate training loads, moved as rapidly as possible (Cormie et al., 2011; Loturco et al., 2015d). In this study, the athletes executed an *optimal loading test* in order to determine their individual *optimal power loads* (i.e., loads capable of generating higher values of muscle power). The maximal JS power has been extensively related to a variety of specific sports measures, including speed and acceleration abilities (Sleivert and Taingahue, 2004; Cronin and Hansen, 2005; Loturco et al., 2015a,b,d). Interestingly, the athletes also presented significant correlations between MPP and actual sprinting time, both in 100 and 200 m dash events (*r* = −0.71 and −0.65, for 100- and 200-m). Of note, the athletes reached their highest MPP at ≈100% of their BM, which represents a substantial amount of external overloading. At this loading condition, movement performance is possibly associated with maximum dynamic strength (i.e., ability to apply high force at low velocity; Baker and Nance, 1999; Young et al., 2001; Stone et al., 2003). Similarly, to start from zero-velocity and achieve higher accelerations in very-short periods, the athletes have to apply greater amounts of force (at lower velocities) against the ground (Wisløff et al., 2004; Loturco et al., 2014). More importantly, based on the parametric relationship between force and velocity (Cronin et al., 2002; Loturco et al., 2015c), these higher rates of acceleration can only be attained if the accelerated mass (i.e., athlete's BM) represents a relatively low resistance for the athlete involved (when compared to his/her maximum dynamic strength; Moss et al., 1997; Cormie et al., 2007; Loturco et al., 2014). Therefore, although we did not have the “partial times” of the actual competitions to perform additional correlational analysis in this study, it is highly conceivable that the more powerful athletes presented superior performance in the acceleration phases of sprinting, in both the 100 and 200 m dash. Undoubtedly, this issue should receive priority in future investigations, in spite of the difficulty in obtaining partial times in official competitions.

In this study, the possibility of combining two independent variables (i.e., SJ and JS) in a multiple linear regression model to more accurately predict the athletes' actual performance was considered due to the distinct importance of each one of these measures in sprinting mechanics and due to the high levels of competitiveness found in Paralympic track and field competitions. As aforementioned, whereas JS is probably more related to the accelerating phases of sprinting (Wisløff et al., 2004; Loturco et al., 2014), SJ may be more associated with the “top-speed” phases of both the 100- and 200-m dash (Loturco et al., 2015b). Therefore, since individual performance in top-level sports depends on very fine adjustments, it is worth considering novel and better models/strategies to predict results and enhance performance. Still, although the use of multiple regression models have increased only (on average) ~1.2% of the explained variance between dependent (actual sprint times) and independent variables (SJ and JS), we considered relevant to carry out this calculation, since slight differences between individuals in 100- and 200-m dash events might significantly affect their competitive results. Observing the data reported here, from this point on, Paralympic coaches can better estimate the actual performance of their top-level athletes in official sprint competitions. Furthermore, by understanding the importance of this specific “mechanical combination” (i.e., SJ and JS) in sprinting performance, they will be able to develop more effective and specific training programs.

Concerning the within-subject variability, the smallest worthwhile enhancements in the 100- (0.9%) and 200-m dash (1.43%) were comparable to those reported in Olympic and Paralympic swimmers (Pyne et al., 2004). However, these values were higher than the estimates provided by elite track athletes without disabilities (Hopkins, 2005). The greater variability in athletes with visual impairment's performances may be associated with their disabilities (Fulton et al., 2009) and the possible influence of their respective guides on the individuals' sprinting mechanics (unpublished data). Importantly, the smallest worthwhile enhancement provides the coach with an idea of the meaningfulness of a given change in an athlete's performance. In general, an enhancement needs to be higher than the smallest worthwhile enhancement to affect the results (i.e., medal prospects; Fulton et al., 2009). Curiously, in the 100-m dash, the athletes presented worse sprinting times than their mean times during the period of observation, but during the Parapan American Games they achieved their “performance peak.” Although in the 200-m the times were close to the mean performance throughout the observation, during the Parapan American Games, they reached a meaningful performance change (in comparison to the previous analyzed competitions). To some extent, this explains the outstanding results obtained by the Brazilian team during the 2015 Pan American Games (three gold, six silver, and two bronze medals in the 100- and 200-m races).

The jumping test results were substantially more variable than the actual competitive performances in the 100- and 200-m dash (CV of SJ = 5.58% and JS = 7.97%), which implies the need for larger improvements in jumping tests, in order to consider these enhancements as meaningful. The general dynamics of the vertical jumping performance were similar to the sprinting performance; nevertheless, only SJ (change > 2.79%) attained a meaningful enhancement prior to the Parapan American Games. Accordingly, while monitoring SJ and JS in Paralympic athletes, coaches need to be aware that the meaningful performance changes should be greater than ≈2.79–3.98%; smaller changes can be considered within the range of the inherent measurement variability. The advantage of these practical and timesaving tests is that they can be easily implemented in the athletes' routines, for finely adjusting the training strategies and, therefore, improving sprinting performance (e.g., stretch-shortening cycle efficiency).

Future studies are necessary in order to determine other factors involved in the non-explained variance of sprinting ability in athletes with visual impairment (e.g., anthropometric characteristics of the subjects and coordination between the athletes with visual impairment and their respective guides, etc). Possibly, some factors related to jumping and running mechanics will reveal more movement similarities and additional associations between their respective kinetic and kinematic parameters.

## Conclusion

Performance in Paralympic elite sprinting depends on a series of neuromuscular, physiological and technical factors. This study showed that vertical jump tests, in loaded and unloaded conditions, could be good predictors of athletes' sprinting performance, mainly when combined in a multiple linear regression equation. Furthermore, in this specific group of top-level Paralympic athletes, the dynamics of SJ and JS performances seem to follow the same variation as 100- and 200-m actual times. These findings may have important implications in athletes with visual impairment's training and testing methodology, since vertical jump tests can be easily performed by subjects with total or partial visual impairments. In addition, Paralympic track and field coaches can use these simple field assessments to specifically monitor their elite athletes close to official competitions, adjusting the training contents in order to optimize each athlete's performance peak. Finally, further studies should be conducted to investigate the chronic effects of training athletes with visual impairment using exclusively loaded and unloaded vertical jumps.

### Conflict of interest statement

The authors declare that the research was conducted in the absence of any commercial or financial relationships that could be construed as a potential conflict of interest.
